# Selenium, Sulfur, and Methyl Jasmonate Treatments Improve the Accumulation of Lutein and Glucosinolates in Kale Sprouts

**DOI:** 10.3390/plants11091271

**Published:** 2022-05-09

**Authors:** Erika Ortega-Hernández, Marilena Antunes-Ricardo, Luis Cisneros-Zevallos, Daniel A. Jacobo-Velázquez

**Affiliations:** 1 Tecnologico de Monterrey, The Institute for Obesity Research, Ave. Eugenio Garza Sada 2501, Monterrey 64849, N.L., Mexico; erika.orhe@gmail.com; 2 Department of Horticultural Sciences, Texas A&M University, College Station, TX 77843, USA; lcisnero@tamu.edu; 3 Tecnologico de Monterrey, The Institute for Obesity Research, Ave. General Ramón Corona 2514, Zapopan 45201, Jal, Mexico

**Keywords:** kale sprouts, glucosinolates, lutein, phenolic compounds, enhancement, sulfur, selenium, methyl jasmonate treatment, antioxidant properties

## Abstract

Kale sprouts contain health-promoting compounds that could be increased by applying plant nutrients or exogenous phytohormones during pre-harvest. The effects of selenium (Se), sulfur (S), and methyl jasmonate (MeJA) on lutein, glucosinolate, and phenolic accumulation were assessed in kale sprouts. Red Russian and Dwarf Green kale were chamber-grown using different treatment concentrations of Se (10, 20, 40 mg/L), S (30, 60, 120 mg/L), and MeJA (25, 50, 100 µM). Sprouts were harvested every 24 h for 7 days to identify and quantify phytochemicals. The highest lutein accumulation occurred 7 days after S 120 mg/L (178%) and Se 40 mg/L (199%) treatments in Red Russian and Dwarf Green kale sprouts, respectively. MeJA treatment decreased the level of most phenolic levels, except for kaempferol and quercetin, where increases were higher than 70% for both varieties when treated with MeJA 25 µM. The most effective treatment for glucosinolate accumulation was S 120 mg/L in the Red Russian kale variety at 7 days of germination, increasing glucoraphanin (262.4%), glucoerucin (510.8%), 4-methoxy-glucobrassicin (430.7%), and glucoiberin (1150%). Results show that kales treated with Se, S, and MeJA could be used as a functional food for fresh consumption or as raw materials for different industrial applications.

## 1. Introduction

Kale (*Brassica oleracea* var. acephala) is a vegetable native to eastern Turkey belonging to the Brassicaceae family [1]. This vegetable has been widely used worldwide in traditional medicine to prevent and treat different health disorders, including cancer, gastric ulcers, high cholesterol levels, hyperglycemia, rheumatism, and hepatic disease, among others [2,3]. Its biological properties have been attributed to different secondary metabolites such as glucosinolates, carotenoid, and phenolic compounds [4,5]. Their beneficial characteristics are based on the scavenging of free radicals, inhibition of receptors, activation of antioxidant enzymes, and variation of gene expression [6]. 

The accumulation of health-promoting bioactive compounds has been related to the application of biotic and abiotic stresses (i.e., wounding, UV radiation, and exogenous phytohormones) in fruits and vegetables through the generation of stress-signaling molecules, and the expression of genes implicated in the secondary metabolism of the plant [7]. Abiotic stresses reported in kale to induce the biosynthesis of glucosinolates, as well as phenolic and carotenoid biosynthesis pathways, include UVA and UVB radiation [8,9,10,11], drought [12,13], sulfur (S) [14,15], selenium (Se) [16], sodium chloride [16,17,18], low and high temperatures [19,20,21,22], and methyl jasmonate (MeJA) [23,24,25].

In plants, S and Se have positive effects, playing a key role in growth and development [26,27]. In tolerable concentrations, S and Se enrich soil nutrients, regulate reactive oxygen species (ROS) levels, and reestablish the cell membrane and chloroplast structures in plants. However, a high concentration of S or Se can trigger the accumulation of ROS [28], which upregulates the expression of several genes involved in phenolic biosynthesis (e.g., the transcription factors *ZmP* and *MYB12,* and their target genes *CHS* and *CHI* –chalcone isomerase) [29]. Moreover, S and Se addition influences the expression of genes involved in the biosynthesis and degradation of glucosinolates (*AT1G16400*) and carotenoids (*PSY* -phytoene synthase) in *Arabidopsis* [30]. Although the influence of S and Se in the Brassicaceae family has been previously reported, research has mainly focused on their effect on mature vegetables and on glucosinolate enhancement [14,15,16], and thus, there is scarce information on its application to enhancing carotenoid and phenolic compound content in kale sprouts. 

On the other hand, phytohormones, such as MeJA are also abiotic stressors that activate physiological and signaling reactions that affect gene expression in plants and result in the production of secondary metabolites [31,32]. In *Arabidopsis* plants, the expression of odorant binding protein (*OBP*) 2 has been shown to be stimulated by MeJA treatment, leading to an induced expression of glucosinolate biosynthetic genes and a subsequent accumulation of glucosinolates [33]. Likewise, it has been confirmed that MeJA treatment can effectively improve the level of total phenolics by increasing phenylalanine ammonia-lyase (PAL) activity in broccoli [34]. On the other hand, little or null change on carotenoid levels due to MeJA application in plants such as kale, broccoli, tomato and lettuce [24,32,35,36] has been reported. However, studies regarding abiotic stresses in kale sprouts with the aim of simultaneously inducing glucosinolate, carotenoid, and phenolic accumulation are very limited, if not non-existent. 

The development of bioaccumulation strategies for nutraceutical compounds in plants, in any of their growth phases, has become a focus of attention in improving the health-benefits of foods and for the design and production of functional ingredients that can potentially be included in various food matrices or even some cosmetics. Therefore, the objective of this study was to determine the individual effects of Se, S and MeJA applied by spraying on the accumulation of glucosinolates, carotenoids, and phenolic compounds in two different varieties of seven-day-old kale sprouts (*Brassica oleracea* var. acephala). 

## 2. Results

### 2.1. Carotenoids

**Figure 1:**
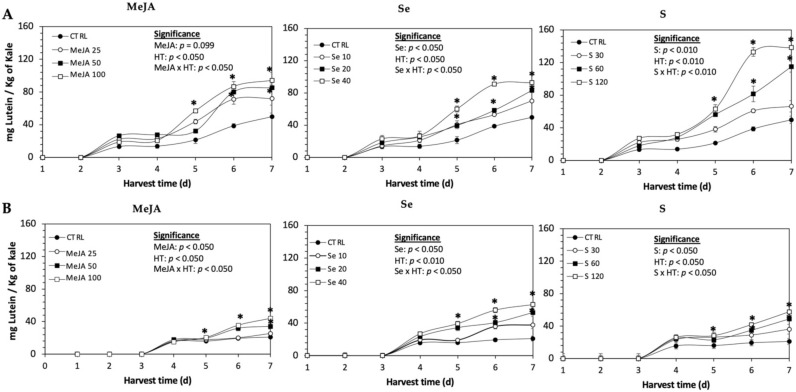
Concentration of lutein in untreated (**A**) Red Russian and (**B**) Dwarf Green kale sprouts and treated with methyl jasmonate, sulfur, and selenium during the first 7 days of germination. Bars are means of 3 replicates ± standard error. Data points with an asterisk (*) indicate statistical difference determined by a *t*-test (*p* < 0.050) between the control and treated samples. Abbreviations: methyl jasmonate (MeJA), sulfur (S), selenium (Se), and harvest time (HT). Results are expressed in dry weight basis.

In plants, carotenoids serve functions in photoprotection, oxidative stress, and developmental regulations, and for humans, they exert antioxidant activity [5]. The main carotenoids reported for kale are lutein and carotene [37,38]. The evaluation of the carotenoid profile of kale sprouts allowed for the identification and quantification of lutein (Figure 1). Values found for lutein were within previously reported ranges for chamber-grown Red Russian (49.8 mg/Kg DW) and Dwarf Green kale (29.0 mg/Kg DW) [37,38,39]. Previous reports have also reported the presence of β-carotene, neoxanthin, and violaxanthin [37,38,40]. The difference in the carotenoid profile reported herein in comparison with previous reports could be attributed to the method of extraction, and the difference in the growing conditions of the other cultivars. 

The daily concentration of lutein during germination (7 d) of kale sprouts treated and non-treated with MeJA, Se or S is shown in Figure 1. The application of MeJA, Se or S did not show an immediate significant change in the content of lutein in any variety of kale. The accumulation of lutein started around 3–4 d in both varieties, without significant differences between treatments. At 5 d of germination, samples showed a significant increase of lutein in both varieties of kale sprouts treated with MeJA 100 µM, Se 40 mg/L or S 120 mg/L. Thereafter, the concentration of lutein increased relatively constantly in all treated samples until the end of germination. The highest increase of lutein was detected in Dwarf Green kale treated with Se 40 mg/L at 7 d of germination, being 199% higher than in the non-treated samples, followed by S 120 mg/L (174%) and MeJA 100 µM (109%). Likewise, lutein significantly increased in Red Russian kale treated with S 120 mg/L (178%), followed by MeJA 100 µM (89%) and Se 40 mg/L (86%).

### 2.2. Glucosinolates

**Figure 2:**
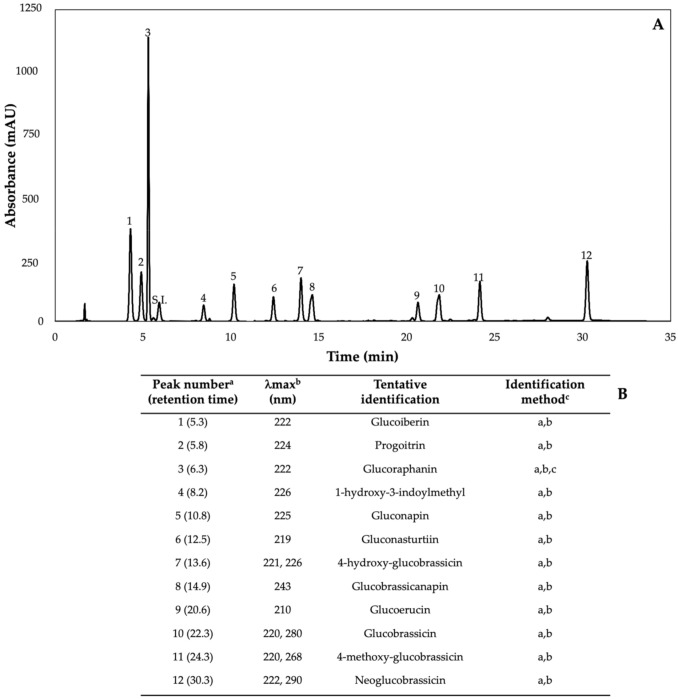
(**A**) HPLC-DAD chromatogram, shown at 227 nm of identified glucosinolates from ethanol/water (70:30, *v*/*v*) extracts of 7-day-old control (water) kale sprouts. (**B**) Tentative identification of individual glucosinolates in kale sprouts was obtained by HPLC-DAD. ^a^ Number of peak assigned according to the order of elution from the C_18_ reverse phase. ^b^ Wavelengths of maximum absorption in the UV/Vis spectra of each chromatographic peak. ^c^ Methods considered for identification of each chromatographic peak: (a) Identification by comparison of UV–Visible spectra and wavelengths of maximum absorbance reported in prior literature; (b) Identification by comparison with the order of chromatographic elution reported by previous authors [32,42,43,44,45,46]; (c) Identification by comparison with the retention time and UV–Visible spectra of authentical standard.

**Figure 3:**
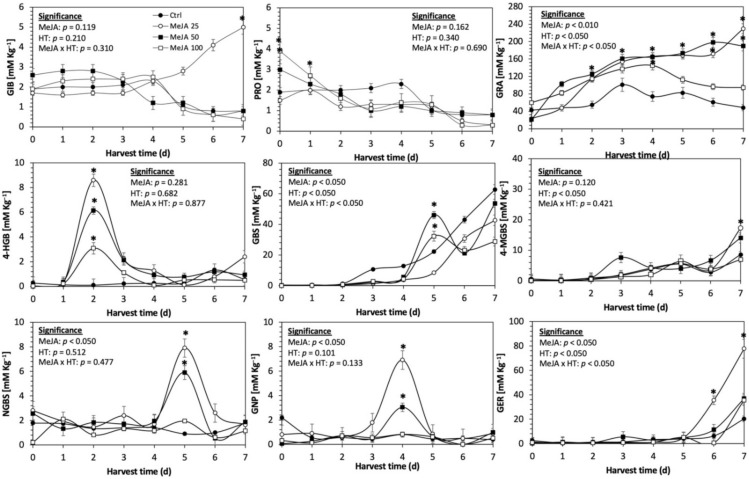
Concentration of individual glucosinolates in untreated Red Russian kale sprouts and treated with methyl jasmonate during the first 7 days of germination. Bars are means of 3 replicates ± standard error. Data points with an asterisk (*) indicate statistical difference determined by a *t*-test (*p* < 0.050) between the control and treated samples. Abbreviations: methyl jasmonate (MeJA), harvest time (HT), glucoiberin (GIB), progoitrin (PRO), glucoraphanin (GRA), 4-hydroxy-glucobrassicin (4-HGB), glucobrassicin (GBS), 4-methoxy-glucobrassicin (4-MGBS), glucoeurocin (GER), gluconapin (GNP), and neoglucobrassicin (NGBS). Results are expressed as desulfogucoraphanin equivalents in dry weight basis.

**Figure 4:**
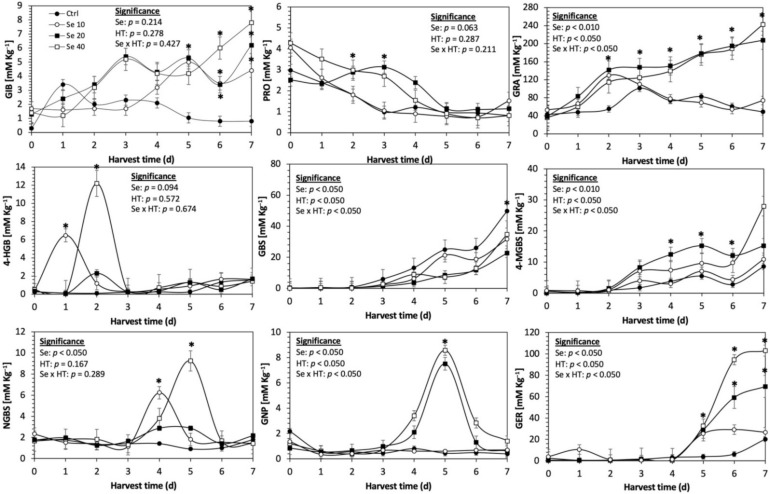
Concentration of individual glucosinolates in untreated Red Russian kale sprouts treated with selenium during the first 7 days of germination. Bars are means of 3 replicates ± standard error. Data points with an asterisk (*) indicate statistical difference determined by a *t*-test (*p* < 0.050) between the control and treated samples. Abbreviations: selenium (Se), harvest time (HT), glucoiberin (GIB), progoitrin (PRO), glucoraphanin (GRA), 4-hydroxy-glucobrassicin (4-HGB), glucobrassicin (GBS), 4-methoxy-glucobrassicin (4-MGBS), glucoeurocin (GER), gluconapin (GNP), and neoglucobrassicin (NGBS). Results are expressed as desulfogucoraphanin equivalents in dry weight basis.

**Figure 5:**
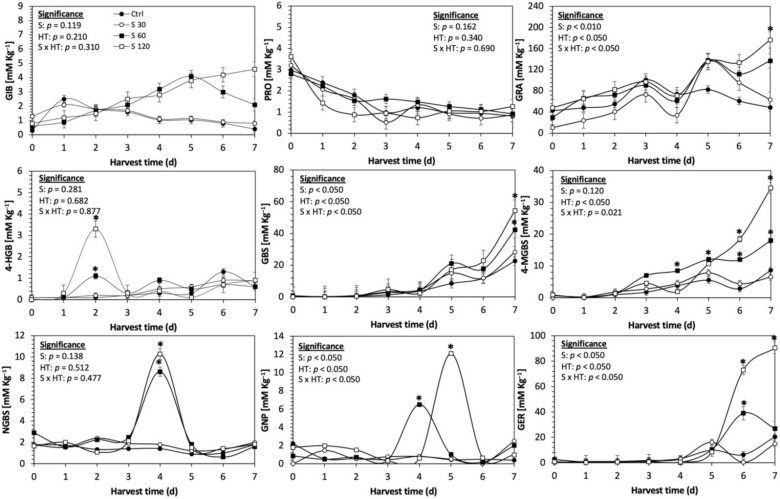
Concentration of individual glucosinolates in untreated Red Russian kale sprouts and treated with sulfur during the first 7 days of germination. Bars are means of 3 replicates ± standard error. Data points with an asterisk (*) indicate statistical difference determined by a *t*-test (*p* < 0.050) between the control and treated samples. Abbreviations: sulfur (S), harvest time (HT), glucoiberin (GIB), progoitrin (PRO), glucoraphanin (GRA), 4-hydroxy-glucobrassicin (4-HGB), glucobrassicin (GBS), 4-methoxy-glucobrassicin (4-MGBS), glucoeurocin (GER), gluconapin (GNP), and neoglucobrassicin (NGBS). Results are expressed as desulfogucoraphanin equivalents in dry weight basis.

**Figure 6:**
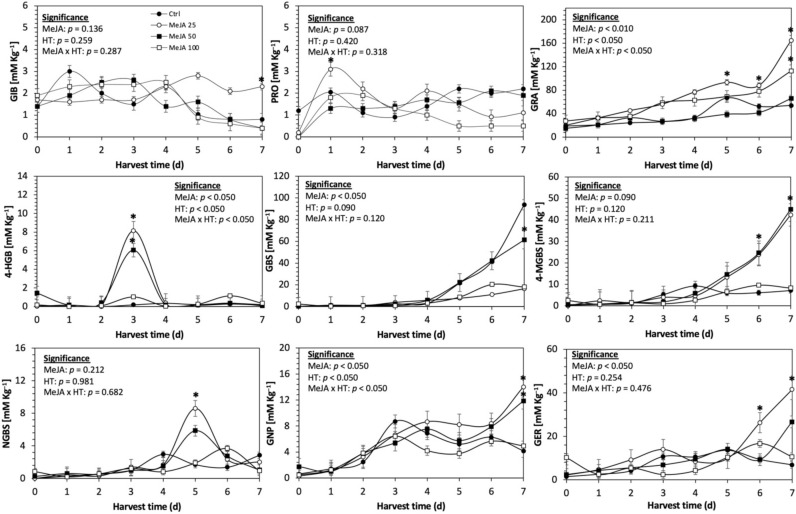
Concentration of individual glucosinolates in untreated Dwarf Green kale sprouts and treated with methyl jasmonate during the first 7 days of germination. Bars are means of 3 replicates ± standard error. Data points with an asterisk (*) indicate statistical difference determined by a *t*-test (*p* < 0.050) between the control and treated samples. Abbreviations: methyl jasmonate (MeJA), harvest time (HT), glucoiberin (GIB), progoitrin (PRO), glucoraphanin (GRA), 4-hydroxy-glucobrassicin (4-HGB), glucobrassicin (GBS), 4-methoxy-glucobrassicin (4-MGBS), glucoeurocin (GER), gluconapin (GNP), and neoglucobrassicin (NGBS). Results are expressed as desulfogucoraphanin equivalents in dry weight basis.

**Figure 7:**
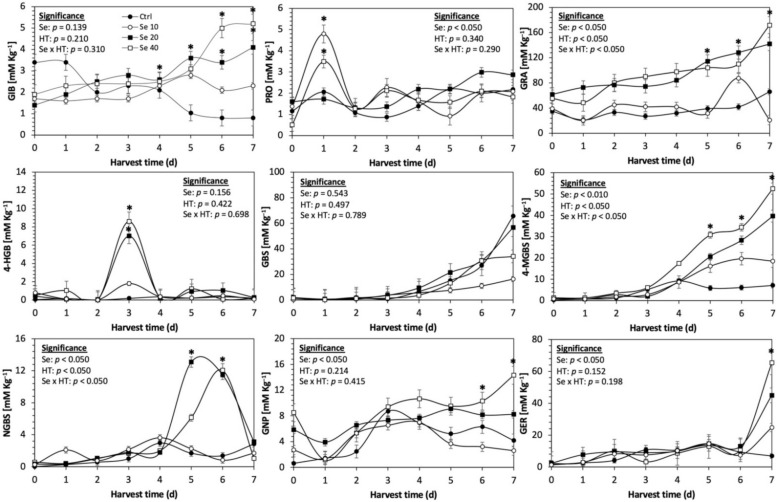
Concentration of individual glucosinolates in untreated Dwarf Green kale sprouts and treated with selenium during the first 7 days of germination. Bars are means of 3 replicates ± standard error. Data points with an asterisk (*) indicate statistical difference determined by a *t*-test (*p* < 0.050) between the control and treated samples. Abbreviations: selenium (Se), harvest time (HT), glucoiberin (GIB), progoitrin (PRO), glucoraphanin (GRA), 4-hydroxy-glucobrassicin (4-HGB), glucobrassicin (GBS), 4-methoxy-glucobrassicin (4-MGBS), glucoeurocin (GER), gluconapin (GNP), and neoglucobrassicin (NGBS). Results are expressed as desulfogucoraphanin equivalents in dry weight basis.

**Figure 8:**
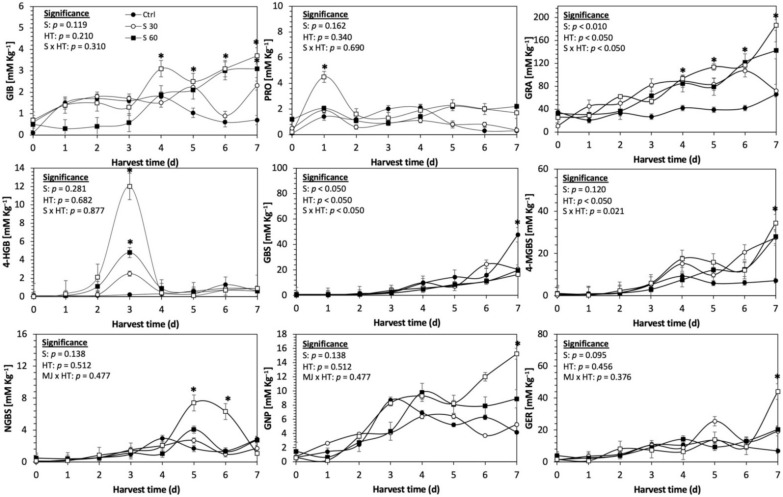
Concentration of individual glucosinolates in untreated Dwarf Green kale sprouts and treated with sulfur during the first 7 days of germination. Bars are means of 3 replicates ± standard error. Data points with an asterisk (*) indicate statistical difference determined by a *t*-test (*p* < 0.050) between the control and treated samples. Abbreviations: sulfur (S), harvest time (HT), glucoiberin (GIB), progoitrin (PRO), glucoraphanin (GRA), 4-hydroxy-glucobrassicin (4-HGB), glucobrassicin (GBS), 4-methoxy-glucobrassicin (4-MGBS), glucoeurocin (GER), gluconapin (GNP), and neoglucobrassicin (NGBS). Results are expressed as desulfogucoraphanin equivalents in dry weight basis.

Glucosinolates are sulfur- and nitrogen-containing thioglucosides biosynthesized from amino acids and glucose [5]. The glucosinolate profile of kale sprouts is shown in Figure 2. Twelve glucosinolates were identified in kale sprouts in both control and treated samples. The glucosinolate profile included six aliphatic glucosinolates: glucoerucin, glucoraphanin, progoitrin, gluconapin, glucoiberin and glucobrassicanapin; one aromatic glucosinolate: gluconasturtiin; and five indolic glucosinolates: glucobrassicin, 1-hydroxy-3-indoylmethyl, neoglucobrassicin, 4-hydroxy-glucobrassicin and 4-methoxy-glucobrassicin. The glucosinolate profile of kale sprouts agrees with previous reports [37,41]. However, the concentration of 9 out of the 12 individual glucosinolates identified varied with the effect of the applied treatment and with harvest time (Figure 3, Figure 4, Figure 5, Figure 6, Figure 7 and Figure 8).

The application of S MeJA 100 µM, Se 40 mg/L, and S 120 mg/L induced an immediate significant increase in the content of glucoiberin (52.9%, 120.3%, and 29.4%) and progoitrin (160%, 72.1%, and 28.5%), respectively, in Red Russian kale sprouts (Figure 3, Figure 4 and Figure 5). Concentrations of glucoiberin and progoitrin in the treated Dwarf Green kale variety remained unaltered compared with control sprouts (Figure 6, Figure 7 and Figure 8). 

The glucosinolate 4-hydroxybrassicin reached its maximum accumulation around day 3–4 of germination in Red Russian and Dwarf green kale sprouts treated with S 120 mg/L (3200% and 3900%), Se 40 mg/L (6000% and 4200%), and MeJA 25 µM (7500% and 3950%), respectively, and its concentration began to decrease in the following days. Later, at 4–5 d of germination, a similar trend was observed in the concentration of the indolyl glucosinolate neoglucobrassicin with S, Se and MeJA treatments. There was a significant increase in neoglucobrassicin concentration by S (635.7% and 335.2%), Se (922.2% and 670.5%), and MeJA (777.7% and 352.6%) in Red Russian and Dwarf green kale sprouts, respectively, followed by a significant decrease. Likewise, the overproduction and subsequent decline of aliphatic gluconapin was also observed in the treated Red Russian kale variety at 4–5 d of germination, whereas in Dwarf green kale, it increased relatively constantly in all treated samples until the end of germination.

In general, the maximum accumulation of glucosinolates was observed in Red Russian kale sprouts harvested at 7 d of germination. The four main glucosinolates overproduced by S 120 mg/L, Se 40 mg/L and MeJA 25 µM stresses were the aliphatic glucosinolates glucoiberin (1150%, 978% and 900%), glucoeurocin (510.8%, 407.8% and 282.2%), and glucoraphanin (262.4%, 397.9% and 371.5%), as well as the indolyl glucosinolate methoxy-glucobrassicin (430.7%, 225.6% and 147.1%) in the Red Russian variety, respectively.

### 2.3. Phenolic Compounds

**Figure 9:**
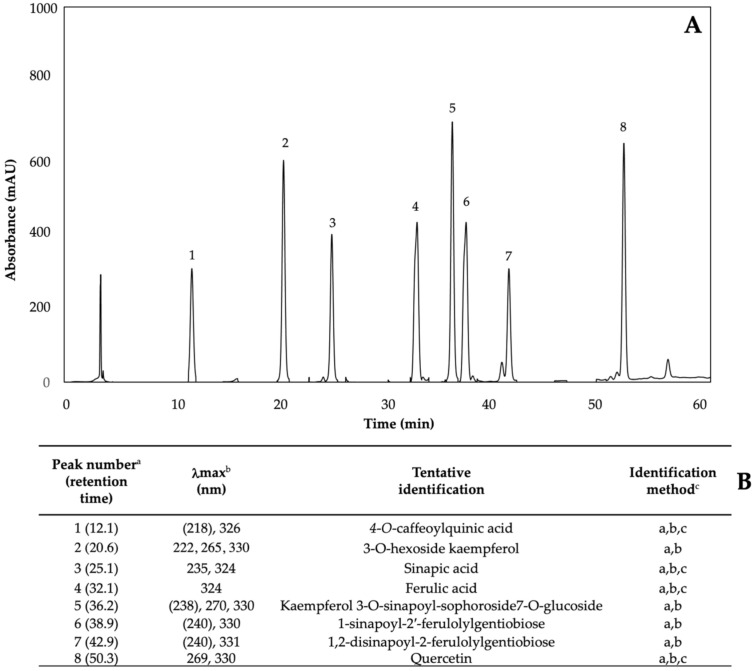
(**A**) HPLC-DAD chromatogram, shown at 320 nm of identified phenolics from ethanol/water (70:30, *v*/*v*) extracts of 7-day-old control (water) kale sprouts. (**B**) Tentative identification of individual phenolics in kale sprouts was obtained by HPLC-DAD. ^a^ Number of peak assigned according to the order of elution from the C_18_ reverse phase. ^b^ Wavelengths of maximum absorption in the UV/Vis spectra of each chromatographic peak. ^c^ Methods considered for identification of each chromatographic peak: (a) Identification by comparison of UV–Visible spectra and wavelengths of maximum absorbance reported in prior literature [5,32,42]; (b) Identification by comparison with the order of chromatographic elution reported by previous authors; (c) Identification by comparison with the retention time and UV–Visible spectra of authentical standard.

Phenolics are the most abundant secondary metabolites in plants, with a structure that possesses an aromatic ring with at least one hydroxyl substituent [5]. The phenolic content of kale sprouts treated with MeJA, Se, and S was also investigated. Eight major phenolic compounds were identified in both control and treated kale sprouts from day 5–7 (Figure 9) including 4-O-caffeoylquinic acid (4-O-CQA); 3-O-hexoside kaempferol (3-O-H-K); sinapic acid; ferulic acid; kaempferol 3-O-sinapoyl-sophoroside 7-O-glucoside (K-3-O-S-so-7-O-g); 1-sinapoyl-2′-ferulolylgentiobiose (1-S-2-FG); 1,2-disinapoyl-2-ferulolylgentiobiose (1,2- diS-2-FG); and quercetin.

Changes in total and individual phenolic content can be observed in Table 1 and Table 2. Most of the phenolic compounds were not detected before day five. Regarding the phenolic content, neither S nor Se caused a significant increase compared to control sprouts; however, all three MeJA (25 µM (−41.3% and −36.3%), 50 µM (−32.3% and −39.4%), and 100 µM (−27.4% and −19.4%)) treatments caused a significant decrease in phenolic concentration for Red Russian (Table 1) and Dwarf Green (Table 2) varieties, respectively, at 7 d of germination. The individual phenolics causing a decrease in overall concentration were sinapic acid; ferulic acid; 4-O-CQA; 1-S-2-FG; and 1,2-diS-2-FG, in both varieties. K-3-O-s-so7-O-g (135.2% and 76.3%) and quercetin (152% and 92.3%) increased significantly in 7-day old Red Russian and Dwarf Green kale sprouts, compared with controls, respectively, with the application of MeJA 25 µM.

### 2.4. Antioxidant Activity

**Figure 10:**
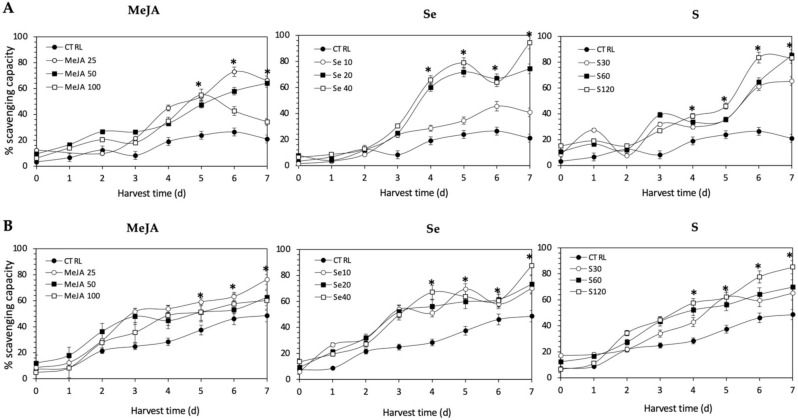
1, 1-diphenyl-2-picrylhydrazyl scavenging activity of ethanolic extract of (**A**) Red Russian and (**B**) Dwarf Green kale sprouts treated with (1) methyl jasmonate, (2) selenium, and (3) sulfur during 7 days of germination. Bars are means of 3 replicates ± standard error. Abbreviations: methyl jasmonate (MeJA), sulfur (S), selenium (Se), harvest time (HT). Data points with an asterisk “*” indicate statistical difference determined by a *t*-test (*p* < 0.050) between the control and treated samples.

The antioxidant activity of all the extracts was determined in terms of the proportion (%) of DPPH scavenged, and values are shown in Figure 10. The application of Se, S or MeJA treatments did not induce an immediate increment in radical scavenging activity in Red Russian and Dwarf Green kale sprouts. After 4 d of germination, significant increments in the DPPH activity of Red Russian and Dwarf Green kale sprouts treated with Se 40 mg/mL (244.3% and 136.2%), S 120 mg/L (100.1% and 102.4%) and MeJA 25 µM (135.8% and 89.4%) were detected, respectively. Moreover, the highest stress-induced antioxidant activity occurred in the Red Russian kale sprouts treated with S 120 mg/L (296.1%) at 7 d, followed by MeJA 25 µM (214.8%) and Se 40 mg/L (93.9%). The maximum percentage of scavenging capacity achieved was 83.5%, 94.3%, and 66.3% in the Red Russian kale sprouts treated with S 120 mg/L, Se 40 mg/L and MeJA 25 µM, respectively, at 7 d.

**Figure 11:**
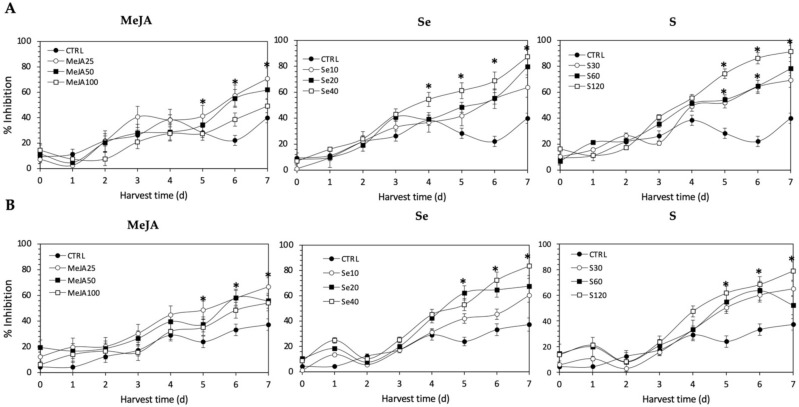
Iron chelating activity of ethanolic extract of (**A**) Red Russian and (**B**) Dwarf Green kale sprouts treated with (1) methyl jasmonate, (2) selenium, and (3) sulfur during the first 7 days of germination. Bars are means of 3 replicates ± standard error. Abbreviations: methyl jasmonate (MeJA), sulfur (S), selenium (Se), harvest time (HT). Data points with an asterisk “*” indicate statistical difference determined by a *t*-test (*p* < 0.050) between the control and treated samples.

Regarding the Fe^2+^-chelating activity (Figure 11), the application of Se, S or MeJA treatments did not induce an immediate increment in kale sprouts. After 5 d of germination, S 120 mg/L (161.9% and 157.8%), Se 40 mg/L (115.7% and 164.7%), and MeJA 25 µM (45.4% and 103.5%) induced a significant increment in Fe^2+^-chelating activity in Red Russian and Dwarf Green kale sprouts, respectively. Fe^2+^-chelating activity increased relatively constantly in all treated samples until the end of germination. Furthermore, the highest increment in chelating activity was observed at 7 d in the Red Russian kale sprouts treated with S 120 mg/L (128.5%), followed by Se 40 mg/L (118.3%) and MeJA 25 µM (77.2%), reaching a percentage of inhibition between 70.8% and 91.3%.

**Figure 12:**
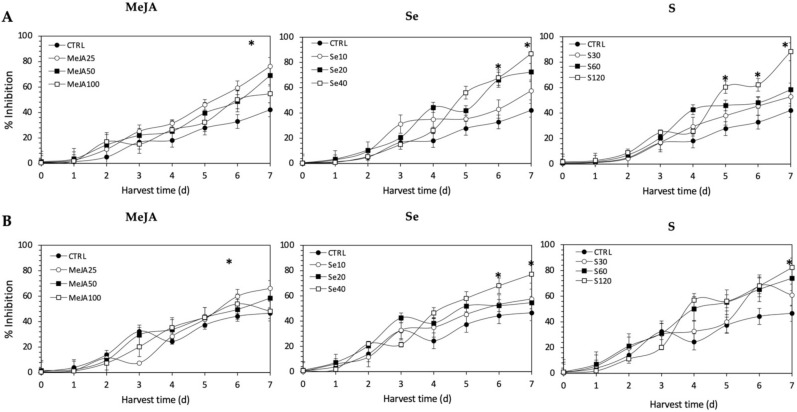
Nitric oxide scavenging activity of ethanolic extract of (**A**) Red Russian and (**B**) Dwarf Green kale sprouts treated with (1) methyl jasmonate, (2) selenium, and (3) sulfur during the first 7 days of germination. Bars are means of 3 replicates ± standard error. Abbreviations: methyl jasmonate (MeJA), sulfur (S), selenium (Se), harvest time (HT). Data points with an asterisk “*” indicate statistical difference determined by a *t*-test (*p* < 0.050) between the control and treated samples.

As observed for radical scavenging and Fe^2+^-chelating activities, the application of Se, S or MeJA treatments did not induce an immediate increment of nitric oxide scavenging activity in kale sprouts (Figure 12). After 4 d of germination, S 120 mg/L (117.1% and 49.7%), Se 40 mg/L (101.6% and 55.28%), and MeJA 25 µM (65.4% and 15.9%) induced significant increments in nitric oxide scavenging activity in Red Russian and Dwarf Green kale sprouts, respectively. Moreover, the highest increment in nitric oxide scavenging activity was observed in the Red Russian kale sprouts treated with S 120 mg/L (109.4%) at 7 d, followed by Se 40 mg/L (106.6%) and MeJA 25 µM (76.3%).

## 3. Discussion

### 3.1. Lutein

The performance of S, Se, and MeJA as abiotic stressors for phytochemical accumulation during the germination of kale was tested in the present study. It was demonstrated that lutein was accumulated in an effective manner with all the evaluated stressors. These results are not consistent with previous reports in kale treated with S or Se, where no differences in carotenoids were found [14,16,42]. In previous research, the carotenoid content in kale leaf (Winterbor, Redbor, and Toscano) showed no response to increasing S levels when supplied at concentrations of 4, 8, 16, 32, and 64 mg of S/L in media [14]. In a similar study, kale leaf lutein accumulation did not respond to treatments with 0–3.5 mg of Se/L [42]. However, these differences could be due to a difference in cultivars used as well as in the growing conditions and growth stage/age of the plant itself. 

Moreover, Sams et al. [30] demonstrated that Se application in *Arabidopsis* can differentially trigger the expression of phytoene synthase (PSY), a major enzyme involved in the biosynthesis of carotenoids. Additionally, it is well known that lutein plays a central role in the deactivation of ^1^O_2_ through complexes of photosystem I and photosystem II [43,44]. Therefore, in the present study, the accumulation of lutein in kale sprouts was probably stimulated to keep ROS levels low and to prevent lipid peroxidation in the thylakoid membrane produced under high levels of Se or S. It is worth mentioning that the current study represents the first attempt to measure the influence of Se on the accumulation of lutein in kale sprouts. Data obtained herein demonstrate that neither S nor Se negatively affected the growth of kale plants within the ranges provided in this study (Supplementary Materials Figures S1 and S2).

Regarding MeJA results, a further increase in lutein content was observed in MeJA 100 µM treatment. Previous studies have reported that exogenous treatment with MeJA has a dose effect on the biosynthesis of carotenoids in maize [45], different varieties of broccoli [46], moringa [47], tomatoes [48], and apples [49]. MeJA has been related to the photosynthetic efficiency and expression of some genes of photosystem II in different cultivars of *Brassica oleracea* L. [46], suggesting that exogenous application of MeJA before germination could influence the assembly, stability, and repair of PS II machinery through the accumulation of carotenoids and sugars. The direct role of MeJA in the enhancement of lutein may be due to the fact that carotenoids are needed to quench several types of pro-oxidants formed continuously during photo-oxidation [50].

On the other hand, there are no previous reports that MeJA application induces lutein accumulation in kale sprouts. MeJA has been used as a stressor in kale close to commercial maturity in higher concentrations (100 µM to 250 µM) with a focus on the induction of other phytochemicals [23,24,25]. Since MeJA plays an important role in plant growth and development [45,46], differences in the results reported herein and previous reports could be attributed to plant variety and maturity stage.

### 3.2. Glucosinolates

The results revealed that the addition of various concentrations of S, Se, and MeJA solution exhibited comparative differences in the glucosinolate contents among the Red Russian and Dwarf Green kale sprouts. The aliphatic glucosinolates were predominant, representing 76.1–87.2% of the total glucosinolate content. 

The present investigation showed that S 120 mg/L was an adequate concentration for inducing the synthesis of glucosinolates in kale. Increments induced by S on total glucosinolate content were mainly attributed to accumulation of the aliphatic glucosinolate glucoraphanin, whereas indolyl 4-methoxyglucobrassicin and glucobrassicin contributed a mean less than 20% of the total glucosinolates. These findings are in agreement with previous reports that indicate that S (2 mM) treatment under greenhouse conditions significantly increased aliphatic glucosinolates and total glucosinolates in ten-week-old kale by 67% and 35%, respectively [15]. Similarly, the application of 32 and 64 mg of S/L has been shown to be effective for inducing the accumulation of glucoiberin, glucobrassicin, neoglucobrassicin, and 4-hydroxygluco-brassicin content in kale leaves [14]. It is proposed that the reason why aliphatic glucosinolates have shown a differential increase in S-treated kale sprouts is that aliphatic glucosinolates are derived from sulfur-containing amino acid methionine, instead of indolic glucosinolates which are synthesized from tryptophan [51].

Moreover, the production of the aliphatic glucosinolates in this study appears to follow patterns based on the R-group and regulation of specific regions of the plant chromosome responsible for the modification of their R-group side chains. The R-groups of glucoiberin and glucoraphanin are both derived from methionine and retain the S atom from methionine. In addition, both are controlled by the same genetic alleles at the Gsl-oxid locus of the plant’s chromosome [52]. The R-groups of gluconapin and progoitrin are also derived from methionine. However, these glucosinolates do not retain the S from methionine, and their final side-chain modification is controlled by alleles at the Gsl-alk and Gsl-oh loci of the plant’s chromosome [52]. On the other hand, the R-group of glucobrassicin, 4-hydroxy-glucobrassicin, and neoglucobrassicin is tryptophan, rather than methionine [52]. This difference in R-group amino acid precursor may help to explain the differences in production of glucobrassicin and neoglucobrassicin, relative to the other glucosinolates analyzed.

Given that Se and S share the same transporters, and the application of Se can influence the expression of genes coding for these proteins, Se uptake was expected to influence glucosinolate concentrations [53,54]. These results agree with Kim et al. [16], who reported that Se (2 mg/L) treatment induces the accumulation of glucosinolates in 6-week-old kale. 

Although the effect of MeJA on glucosinolate accumulation during kale germination has not been characterized, treatment with MeJA 250 μM has been demonstrated to be successful in inducing the accumulation of total glucosinolates in broccoli sprouts [55]. In support of this argument, treatment with MeJA may stimulate the expression of genes associated with the biosynthesis of glucosinolates [56,57]. 

Compared to S and Se supplementation, a smaller increase in glucosinolate content was observed with MeJA treatment. However, a similar trend was observed in the proportion of the indolyl and aliphatic glucosinolates, with glucoiberin, glucoeurocin, glucoraphanin, glucobrassicin, and methoxy-glucobrassicin being overproduced. These results are in agreement with Moreira-Rodríguez et al. [32] and Mikkelsen et al. [56], who suggest that MeJA treatment induces the flux of carbon from indolic glucosinolates to aliphatic glucosinolates in the biosynthesis pathway. Moreira-Rodríguez et al. [32] and Wisner et al. [58] reported a significant increase in glucoraphanin in sprouts of broccoli and pak choi treated with MeJA 25 μM and 200 μM, respectively. An induction of transcription factors (i.e., *MYB51*, *MYB34*, and *OBP2*) involved in the biosynthesis of indolic glucosinolates has been reported in the presence of MeJA treatment [33].

Regarding indolic synthesis, it has been reported that glucobrassicin is synthesized by enzymes sulfotransferases 16 and 18, and is then converted into 1-hydroxy-3-indoylmethyl or 4-hydroxybrassicin by hydroxylation reactions by the enzymes CYP81F1–3 and CYP81F4, respectively. Then, these glucosinolates are methylated to neoglucobrassicin and 4-methoxyglucobrassicin by 2-O-methyltransferases, respectively [58]. Therefore, the observations presented here suggest that the application of MeJA treatment stimulates the methylation of glucobrassicin and that such an effect involves the formation of highly unstable intermediates, i.e., neoglucobrassicin and 4-methoxyglucobrassicin.

### 3.3. Phenolic Compounds

The evaluation of Se and S on phenolic acids in kale sprouts showed that it may not be profitable or even possible to enhance the content of multiple phytochemicals of a single plant. Since Se and S follow similar metabolic routes, the opposing response between glucosinolates and phenolics may be related to interference in carbon assimilation pathways, or alternatively favoring one pathway over the other. In agreement with our results, Finley et al. [59] have reported inhibition of phenolic acids in broccoli in the presence of Se. 

Furthermore, Moreira-Rodríguez [32] and Villareal-García [60] have also reported a decrease in the accumulation of specific phenolic compounds due to MeJA-spraying in broccoli including 1-S-2-FG, 1,2,2-triSG, 1,2-diS-2-FG, and 4-O-CQA. Previous works have shown that MeJA and JA treatments may prevent lignification in a concentration-dependent manner [61] and may downregulate genes involved in the biosynthesis of phenolics and lignification (PAL, 4-coumarate-CoA ligase (4CL), and caffeoyl-CoA 3-O-methyltransferase (CCoAOMT)) [62]. Additionally, a large increase in kaempferol and quercetin compounds with MeJA 25 µM suggests a redirection of the carbon source towards the biosynthesis of flavonoids. 

Although glucosinolates and phenylpropanoids are synthesized through distinct biosynthetic pathways, it has been confirmed that indole glucosinolate biosynthesis limits phenylpropanoid accumulation in *Arabidopsis* due to an overproduction of the substrate indole-3-acetaldoxime [32,63]. This is an intermediate in tryptophan-derived glucosinolate biosynthesis which limits phenylpropanoid accumulation mainly through decreased PAL activity and substrate availability [63].

### 3.4. Antioxidant Activity

Due to the complex nature of phytochemicals and their mechanisms of action, no single testing method can provide a full representation of the antioxidant profile. The antioxidant capacities of the treated and control ethanolic kale extracts were determined by measuring free radical scavenging activity, chelating activity, and nitric oxide assays.

All the antioxidant activity determination methods used in this study showed the same trends, with the content of glucosinolates and lutein differentially stimulated by the different treatments. The antioxidant activities showed an increasing trend in all methods as the concentrations of the extracts increased. The highest scavenging capacity was recorded from Se 40 mg/L-treated kale sprouts after 7 d of germination (94.3%), followed by S 120 mg/L (83.4%) and MeJA 25 µM (66.3%). Likewise, the highest percentage of the inhibition of formation of the Ferrozine-Fe^2+^ complex, around 91.3%, was found with S 120 mg/L-treated Red Russian kale sprouts at 7 d of germination, followed by Se 40 mg/L (87.3%) and MeJA 25 mg/L (70.8%). On the other hand, the extracts from treated kale were checked for their inhibitory effects on nitric oxide release from sodium nitroprusside. The highest percentage of inhibition, 88.2%, was found with S 120 mg/L-treated Red Russian kale sprouts at 7 d of germination, followed by Se 40 mg/L (87.0%) and MeJA 25 µM (76.3%).

The bioactivity of glucosinolates and their hydrolysis products have been reported in the Brassicaceae family [64,65,66,67]. Although the direct antioxidant action of glucosinolates is controversial, due to the fact that their antioxidant activity is mainly associated with transcription triggering of phase II metabolic enzymes [68], the direct antioxidant potential of glucoerucin in arugula extract (*Eruca sativa*) [69], glucobrassicin in cauliflower (*Brassica oleracea* L.) [70], and glucoraphasatin in radish (*Raphanus sativus* L.) [71] has been demonstrated. Some authors have proposed that the antioxidant capacities of glucosinolates is quite specific and has a structural explanation. The sulfur atom in the methyl thiol group present in the side chain of some glucosinolates can act as an electron donor, switching from a reduced form (the sulphide group) to an oxidized form (the sulphinyl group), generating redox pairs [69,71]. Moreover, the indole glucosinolates, such as glucobrassicin, probably act as proton donors, whereas gluconasturtiin possesses metal-ion-chelating activity [72]. In addition, it is important to consider that the in vivo antioxidative potential of glucosinolates is greatly increased by myrosinase-mediated breakdown to highly active derivatives.

Likewise, the results obtained from antioxidant activity assays of ethanolic kale extracts correlate with the response of lutein content in the samples. It is known that lutein is a pigment which plays a major role in the protection of plants against photooxidative processes [73]. Like other carotenoids, lutein has the ability to quench singlet oxygen and scavenge toxic free radicals through its double carbon–carbon bonds and cyclic substituents [74]. In agreement with these results, the influence of lutein in the antioxidant activity of kale has also been previously reported by Ligor et al., [75] and Sikora & Bodziarczyk [76].

Although there was no significant increase in the content of most phenolic compounds (except for kaempferol and quercetin) related to the different treatments applied, their intrinsic levels in kale may possibly contribute to antioxidant activity, likely through an interaction (antagonistic, additive or synergistic) with the other bioactive molecules. It has been reported that the antioxidant capacity of phenolic compounds is mainly attributed to their methoxy, hydroxyl, and carboxylic acid groups [77], which can play an important role in neutralizing free radicals, quenching singlet and triplet oxygen, or decomposing peroxides [78]. Furthermore, the antioxidant properties of polyphenol extracts in several Brassica plants such as broccoli [79], kale [76], and *B. incana* [80] have been confirmed. Thus, the overall antioxidant activity can be the result of the combination of all three induced phytochemicals, where the induced contents of glucosinolates >> phenolics > lutein.

## 4. Materials and Methods

### 4.1. Chemicals

Methyl jasmonate (MeJA); sulfatase (from *Helix pomatia*); diethylaminoethyl (DEAE)-sephadex A-25; sinigrin hydrate; sodium acetate; ferrozine (3-(2-Pyridyl)-5,6-diphenyl-1,2,4-triazine-p,p′-disulfonic acid monosodium salt hydrate); iron (II) sulfate heptahydrate (FeSO_4_·7H_2_O); DPPH (1,1-diphenyl-2-picrylhydrazyl); sodium nitroprusside; acetonitrile (HPLC grade); methanol (HPLC grade); methyl tert-butyl ether (MTBE; HPLC grade); phosphoric acid; 4-*O*-caffeoylquinic acid; ferulic acid; sinapic acid; quercetin; kaempferol and lutein standards were obtained from Sigma Chemical Co. (St. Louis, MO, USA). Sulfanilamide and N-(1-Naphthyl)ethylenediamine dihydrochloride (NED) solutions were acquired from Promega (G2930, Madison, WI, USA). Ethanol (HPLC grade), sodium selenite (Se; Na_2_SeO_3_), and potassium sulfate (S; K_2_SO_4_) were obtained from Desarrollo de Especialidades Quimicas, S.A. de C.V. (Monterrey, NL, Mexico). Desulfoglucoraphanin was acquired from Santa Cruz Biotechnology (Dallas, TX, USA).

### 4.2. Plant Material

Red Russian and Dwarf Green kale (*Brassica oleracea* var. acephala) seeds were provided by La Semilleria (Queretaro, Qro, Mexico). Kale seeds were sanitized for 15 min in sodium hypochlorite (1.5%, *v/v*) and rinsed with Milli-Q water. 

### 4.3. MeJA, Se, and S Treatments 

Kale seeds were soaked in Milli-Q water (control) or the treatment solutions for 5 h. The treatment solutions used were Se (10, 20, 40 mg/L), S (30, 60, 120 mg/L), and MeJA (25, 50, 100 µM). Afterwards, the soaking solutions were discarded, and the seeds were placed on germination trays in a dark chamber set at 25 °C and 85% of relative humidity for 7 days. Three replicates of each treatment were run concurrently. Each replica consisted of a tray containing 30 g of kale seeds sprayed with 5 mL of different treatment concentrations or control every 12 h throughout the experiment. Samples were collected every 24 h for 7 days, freeze-dried (Labconco, Kansas City, MO, USA), ground into a powder, and stored at −80 °C until further analysis of phytochemicals.

### 4.4. Phytochemical Analyses

#### 4.4.1. Extraction of Secondary Metabolites

Extraction of glucosinolates and lutein from kale sprouts powder was carried out in a single process based on the protocol published by Moreira-Rodríguez et al. [32]. Briefly, kale samples (0.2 g) were mixed with 10 mL of ethanol/water (70:30, *v*/*v*), previously heated for 10 min at 70 °C to inactivation of myrosinase, followed by the addition of 50 μL of sinigrin 3 mM as internal standard (I.S.).

Samples were immersed in a water bath (VWR, Radnor, PA, USA) at 70 °C and vortexed every 5 min for 30 min to inactivate myrosinase. Then, the extracts were left to cool at 25 °C and centrifuged (18,000× *g*, 10 min, 4 °C) (SL16R, Thermo Scientific, GER) to recover the supernatant. The supernatant obtained was used for the identification and quantification of individual glucosinolates, lutein, and phenolic compounds by HPLC-DAD (1260 Infinity, Agilent Technology, Santa Clara, CA, USA).

#### 4.4.2. Identification and Quantification of Lutein 

The identification and quantification of lutein were performed as described by Moreira-Rodríguez et al. [32]. Briefly, 25 µL of clarified ethanolic extracts, previously filtered using 0.45 µm nylon membranes (VWR, Radnor, PA), were injected in the HPLC-DAD system (1260 Infinity, Agilent Technologies, Santa Clara, CA). Lutein was separated on a YMC C30 column (4.6 mm × 150 mm, 3 µm particle size) (YMC, Wilmington, NC, USA). The mobile phase consisted of (A) methanol, (B) MTBE, and (C) water. The elution system was isocratic: 50% A, 45% B, and 5% C for 30 min with a flow rate of 0.5 mL/min. Lutein was detected at 450 nm and identified by comparing its retention time and absorption spectra with a reference standard. Lutein was quantified using a calibration curve of lutein standard (0–12 ppm) and expressed as mg of lutein per kg of kale sprouts in dry weight (DW) basis. 

#### 4.4.3. Identification and Quantification of Phenolic Compounds

Phenolic compounds in kale extract were quantified using an HPLC system (Agilent Technologies 1260 series, Santa Clara, CA, USA) coupled with a diode array detector (DAD). The stationary phase was a C18 reverse phase column, 4.6 mm × 250 mm, 5 μm (Luna, Phenomenex, Torrance, CA, USA). Two mobile phases were used for chromatographic separation: (A) water adjusted to pH 2.4 with phosphoric acid and (B) methanol-water (60:40, *v*/*v*). Gradient elution consisted of 0/0, 3/30, 8/50, 35/70, 40/80, 45/100, 50/100 and 60/0 (min/% phase B) at a flow rate of 0.8 mL/min^.^ The injection volume was 10 μL. The analysis was performed at 280, 320, and 360 nm and integrated by the OpenLAB CDS ChemStation software (Agilent Technologies, Santa Clara, CA, USA). 

Peak identification was based on retention time, UV-visible spectra and wavelengths of maximum absorption, as compared with the reported literature [32,81] and commercial standards. For the quantification of phenolic compounds, standard curves of 4-*O*-caffeoylquinic acid (0.1–50), ferulic acid (0.4–40 ppm), sinapic acid (0.1–50 ppm), quercetin (0.1–12 ppm), and kaempferol (0.1–12 ppm) were prepared. Results were expressed as mg of each individual phenolic compound per Kg of kale sprouts (mg/Kg) in dry weight (DW) basis.

#### 4.4.4. Desulfation of Glucosinolates

After extraction, a desulfation step was carried out to purify glucosinolates and improve accuracy and identification from HPLC. DEAE-Sephadex A-25 resin was hydrated for at least 12 h in sodium acetate (0.02 M, pH 5). Polypropylene syringes were washed with 0.5 mL of water, and 0.5 mL of hydrated DEAE-sephadex A-25 was added, followed by an additional wash with 0.5 mL of water. Sephadex was left to pack and the excess medium removed after 20 min. Then, columns were loaded immediately with 3 mL of kale supernatant. After removing excess supernatant by elution, the columns were washed with 1 mL of water followed by 1 mL of sodium acetate (0.02 M). Purified sulfatase (75 μL) was added to each column and was left at 25 °C for 12 h. Desulfoglucosinolates were collected in vials by elution, slowly adding 1.25 mL of water.

#### 4.4.5. Identification and Quantification of Glucosinolates

Glucosinolates were identified and quantified by HPLC-DAD. Separation was performed on a C18 reverse phase column, 4.6 mm × 250 mm, 5 μm (Luna, Phenomenex, Torrance, CA, USA). Elution was conducted with (A) water and (B) acetonitrile. Separation was achieved with an initial A concentration of 100%, adjusting the A concentration to 80% at 28 min and to 100% at 35 min at a flow rate of 1.5 mL/min. The injection volume was 20 μL. Data were acquired at 227 nm and processed by the OpenLAB CDS ChemStation software (Agilent Technologies, Santa Clara, CA, USA). 

Glucosinolate identification was based on retention time, UV-visible spectra and wavelengths of maximum absorption as compared with the reported literature [32,81] and commercial standards. For the quantification of glucosinolates, a standard curve of desulfoglucoraphanin (0–1250 ppm) was prepared. Results were expressed as mmol of desulfoglucoraphanin equivalents per Kg of kale sprouts (mM/Kg) (DW).

### 4.5. Antioxidant Activities

#### 4.5.1. Free Radical Scavenging Activity

The free radical scavenging activity of ethanolic kale extracts was determined using the DPPH method proposed by Miceli et al. [82]. Briefly, an aliquot (20 µL) of extract of kale was added to 280 µL of daily prepared methanol DPPH solutions (0.1 mM). The solution was incubated for 30 min in the absence of light. Finally, the optical density change at 517 nm was measured. The scavenging activity was measured as the decrease in absorbance of the samples versus DPPH standard solution. The results were reported as mean radical scavenging activity percentage (%) ± SD. The radical scavenging activity percentage (%) of the DPPH was calculated by Equation (1):

(1) $$\%\;of\;scavenging\;activity=\left[\frac{Ac-As}{Ac}\right]\times 100$$

where *Ac* is the absorbance of the control and *As* is the absorbance in the presence of the sample. 

#### 4.5.2. Ferrous Ion (Fe^2+^)-Chelating Activity

The Fe^2+^-chelating activity of the kale extracts was evaluated using the method described by Vavrusova et al. [83]. An aliquot (25 µL) of the kale extract diluted to 500 µg/mL was mixed with 100 µL of FeSO_4_ (75 µM). The reaction was initiated by the addition of 100 µL of ferrozine (500 µM). Then the mixture was shaken vigorously and left to stand at room temperature for 10 min. The absorbance of the solution was measured spectrophotometrically at 562 nm. The percentage of inhibition of ferrozine–(Fe^2+^) complex formation was calculated using equation 1, where *Ac* is the absorbance of the control (FeSO_4_ and ferrozine) and *As* the absorbance in the presence of the sample. 

#### 4.5.3. Nitric Oxide Radical Assay

The scavenging of nitric oxide radicals capacity of the kale sprout extracts was determined using the procedure described by Hazra et al. [84]. Sodium nitroprusside (100 μL, 10 mM) in standard phosphate buffer saline (pH 7.4) was incubated with 200 μL of kale extract (500 µg/mL) at 25 °C for 2 h 30 min. After incubation, samples (50 μL) were diluted with 25 μL of sulfanilamide. The sample was incubated for 5 min at room temperature. Then, 25 μL of NED was added and the assay was left standing for 30 min at room temperature. The absorbance was observed at 540 nm on a spectrophotometer. The percentage of inhibition was calculated according to equation 1.

### 4.6. Statistical Analysis

Statistical analysis analyses were carried out using three replicates. Results were obtained as mean values and their standard error. Statistical analyses were performed using JMP software version 13.0 (SAS Institute Inc., Cary, NC). Data were analyzed by full factorial analyses of variance (ANOVA) followed by the least significant difference (LSD) test (*p* < 0.05). 

## 5. Conclusions

The recent identification of kale sprouts as a rich source of phytochemicals with anti-inflammatory, anti-cancer, and antioxidant attributes has directed scientific efforts towards improving its content. Results obtained herein demonstrate that MeJA, Se, and S treatments can be used as simple steps during germination to enhance levels of specific secondary plant metabolites including glucosinolates and lutein.

Dwarf Green kale treated with Se 40 and harvested after 7 d resulted in the highest increase of lutein, followed by Red Russian treated with S 120 and harvested after 7 d. 

In addition, S 120 could be used as an effective treatment for inducing the accumulation of total and individual glucosinolates during the first 7 d of germination of kale sprouts. Treatments led to the accumulation of aliphatic and indolyl glucosinolates in both varieties; however, S and Se favored mainly the accumulation of GRA, GER, 4-MGBS, and GIB, whereas the latter favored overproduction of 4-MGBS, GIB, and GBS. 

Data herein presented suggest that the phytochemicals accumulated as a stress response are supported mainly in a complex carbon flux model acting on the biosynthesis pathway of specific glucosinolates. It is important to study the effects of these stresses on other varieties of kale to study their behavior due to differences in phytochemical profiles and required growing conditions. 

The stressed kale sprouts with enhanced levels of glucosinolates and lutein could be used as foods or as raw materials to produce nutraceutical foods, dietary supplements, or cosmeceuticals. Moreover, stressed kale sprouts could be subjected to downstream processing to extract and purify the phytochemicals accumulated, which can be further commercialized as high-value, health-promoting biomolecules. In order to commercialize kale sprouts treated with MeJA, Se, and S as ready-to-eat food with enhanced levels of glucosinolates and lutein, further experiments should be performed to determine shelf-life stability and to validate the bioactivity of the enhanced phytochemicals. Moreover, further experiments should also determine the accumulation of Se and S in the sprouts to determine their levels and ensure their safety before commercialization.

## Figures and Tables

**Table 1:** Concentration of individual and total phenolic compounds in Red Russian kale sprouts treated with MeJA, Se, and S after 7 d of germination.

Phenolic Compound Concentration (mg/100 g DW) ^1,2,3^																																				
Treatment	4-O-CQA				3-O-H-K				Sinapic acid				Ferulic acid				1-S-2-FG				1,2-diS-2-FG				K-3-O-s-so7-O-g				Quercetin				Total			
CTRL	47.69	±	0.65	a	80.12	±	2.77	a	68.27	±	1.12	a	21.59	±	0.96	a	4.00	±	0.84	a	29.37	±	1.26	a	27.10	±	1.04	c	12.71	±	0.66	bc	290.84	±	9.31	a
MeJA 25 µM	18.68	±	0.97	c	17.52	±	5.61	d	18.48	±	2.82	e	9.53	±	0.26	c	1.42	±	0.58	c	10.29	±	2.28	b	63.67	±	1.85	a	32.02	±	1.48	a	171.59	±	15.85	e
MeJA 50 µM	29.88	±	0.85	bc	72.24	±	4.61	a	26.76	±	2.25	d	6.20	±	0.90	c	0.76	±	0.10	d	15.37	±	2.29	b	34.81	±	0.36	b	11.47	±	0.40	bc	197.77	±	11.77	d
MeJA 100 µM	33.01	±	0.96	b	62.99	±	5.44	b	39.23	±	3.47	c	6.09	±	0.74	c	1.51	±	0.64	c	12.04	±	0.85	b	31.19	±	1.51	b	22.95	±	2.80	b	212.31	±	16.40	d
Se 10 mg/L	36.43	±	1.09	b	61.74	±	0.62	b	24.95	±	1.38	d	17.15	±	0.97	ab	3.84	±	0.13	a	24.18	±	0.90	a	17.65	±	1.67	d	14.93	±	0.77	b	200.87	±	7.53	d
Se 20 mg/L	39.61	±	2.13	b	66.13	±	4.13	b	27.22	±	2.73	c	18.25	±	0.93	ab	3.62	±	0.13	a	24.36	±	0.50	a	26.03	±	1.69	c	23.75	±	1.57	b	228.97	±	13.81	d
Se 40 mg/L	33.69	±	2.69	b	49.65	±	2.23	c	32.60	±	1.56	c	23.92	±	1.40	a	3.98	±	0.29	a	25.61	±	1.85	a	19.35	±	0.92	d	26.86	±	0.57	b	215.67	±	11.51	d
S 30 mg/L	46.69	±	1.32	a	71.41	±	3.68	ab	51.36	±	6.27	b	16.15	±	0.87	ab	3.20	±	0.05	a	26.00	±	0.44	a	21.78	±	1.57	d	13.54	±	0.12	bc	250.12	±	14.33	c
S 60 mg/L	41.33	±	3.7	a	86.14	±	4.13	a	73.22	±	3.28	a	19.25	±	0.86	a	2.95	±	0.85	ab	27.13	±	0.60	a	26.69	±	1.28	b	19.76	±	1.62	b	296.46	±	16.32	a
S 120 mg/L	32.83	±	6.68	b	79.36	±	2.23	a	58.6	±	5.27	b	17.92	±	1.69	a	3.23	±	0.51	a	26.63	±	1.01	a	39.73	±	0.06	b	21.08	±	0.49	b	279.39	±	17.94	b

^1^ Concentrations are reported as each individual standard. Compounds were quantified at 320 nm: sinapic acid; ferulic acid; 4-O-caffeoylquinic acid (4-O-CQA); 1-sinapoyl-2′-ferulolylgentiobiose (1-S-2-FG); and 1,2-disinapoyl-2-ferulolylgentiobiose (1,2- diS-2-FG); 360 nm: kaempferol 3-*O*-sophoroside-7O-glucoside (K-3-O-s-so7-O-g), 3-O-hexoside kaempferol (3-O-H-K), and quercetin. ^2^ Values represent the mean of three replicates ± standard error of the mean. ^3^ Different letters in the same column indicate statistical differences in the concentration of each compound between treatments using the least significant difference (LSD) test (*p* < 0.05). Concentrations of K-3-O-s-so7-O-g and 3-O-H-K are expressed as kaempferol equivalents; concentrations of 1-S-2-FG and 1,2-diS-2-FG are expressed as ferulic acid equivalents. Authentic standards were used to determine the concentrations of 4-O-CQA, sinapic acid, ferulic acid, and quercetin.

**Table 2:** Concentration of individual and total phenolic compounds in Dwarf Green kale sprouts treated with MeJA, Se, and S after 7 d of germination.

Phenolic Compound Concentration (mg/100 g DW) ^1,2,3^																																				
Treatment	4-O-CQA				3-O-H-K				Sinapic acid				Ferulic acid				1-S-2-FG				1,2-diS- 2-FG				K-3-O-s-so7-O-g				Quercetin				Total			
CTRL	43.34	±	2.90	a	91.18	±	4.16	a	84.68	±	4.02	a	28.04	±	1.11	a	6.32	±	0.42	a	3.51	±	2.95	ab	32.86	±	2.36	cd	23.97	±	0.99	c	313.91	±	18.89	a
MeJA 25 µM	17.16	±	0.97	d	32.52	±	3.03	d	28.48	±	2.22	e	13.96	±	0.26	c	2.42	±	0.83	c	0.93	±	2.28	c	57.92	±	1.85	a	46.10	±	1.72	a	199.96	±	13.16	d
MeJA 50 µM	25.88	±	0.85	c	40.45	±	7.61	c	36.27	±	2.11	e	12.20	±	0.48	c	2.52	±	0.77	c	0.37	±	0.97	c	41.98	±	0.10	b	31.11	±	0.31	b	190.23	±	13.2	d
MeJA 100 µM	29.32	±	0.49	c	67.23	±	3.35	b	46.97	±	4.22	d	11.97	±	1.94	c	2.35	±	0.83	c	1.30	±	0.56	c	41.19	±	1.23	b	26.95	±	1.61	bc	227.90	±	14.22	c
Se 10 mg/L	39.98	±	1.24	a	72.10	±	6.68	b	62.34	±	3.27	c	34.28	±	0.97	a	6.71	±	0.05	a	4.76	±	0.39	a	16.88	±	1.57	d	27.00	±	0.01	bc	264.03	±	14.18	b
Se 20 mg/L	31.13	±	2.70	b	74.35	±	7.30	b	72.04	±	3.28	b	28.50	±	0.93	a	5.58	±	0.82	b	3.13	±	0.03	ab	28.87	±	1.03	cd	30.75	±	1.62	b	274.35	±	17.72	b
Se 40 mg/L	38.32	±	2.07	ab	94.36	±	5.33	a	69.11	±	4.33	c	23.89	±	1.40	ab	4.56	±	0.61	b	4.86	±	0.50	a	24.32	±	0.21	d	22.86	±	0.90	c	282.28	±	15.34	ab
S 30 mg/L	40.98	±	1.37	a	99.99	±	7.60	a	73.74	±	5.96	a	27.83	±	0.87	a	7.76	±	0.40	a	4.56	±	0.85	a	19.32	±	1.92	d	18.93	±	0.98	c	293.11	±	19.95	a
S 60 mg/L	33.65	±	2.21	b	75.70	±	7.33	b	88.00	±	4.88	a	32.82	±	0.86	a	8.13	±	0.62	a	3.30	±	0.10	ab	34.97	±	1.73	c	21.75	±	1.79	c	298.32	±	19.53	a
S 120 mg/L	39.89	±	2.47	a	92.99	±	9.96	a	74.11	±	7.44	a	24.95	±	1.69	ab	9.84	±	0.78	a	4.60	±	1.51	b	35.63	±	0.84	c	27.58	±	0.98	bc	309.59	±	25.68	a

^1^ Concentrations are reported as each individual standard. Compounds were quantified at 320 nm: sinapic acid and ferulic acid, 4-O-caffeoylquinic acid (4-O-CQA), 1-sinapoyl-2′-ferulolylgentiobiose (1-S-2-FG) and 1,2-disinapoyl-2-ferulolylgentiobiose (1,2-diS-2-FG); 360 nm: kaempferol 3-*O*-sophoroside-7O-glucoside (K-3-O-s-so7-O-g), 3-O-hexoside kaempferol (3-O-H-K) and quercetin. ^2^ Values represent the mean of three replicates ± standard error of the mean. ^3^ Different letters in the same column indicate statistical differences in the concentration of each compound between treatments using the least significant difference (LSD) test (*p* < 0.05).

## Data Availability

The data presented in this study are available within the article.

## Supplementary Materials

The following supporting information can be downloaded at: https://www.mdpi.com/article/10.3390/plants11091271/s1, Figure S1: Seed germination of Red Russian and Dwarf Green kale (*Brassica oleracea* var. acephala) treated with methyl jasmonate, selenium, sulfur or water as control for 7 days; Figure S2: Comparison of Red Russian (left) and Dwarf Green kale (right) sprouts (*Brassica oleracea* var. acephala) germinated with most significant treatments: methyl jasmonate 25 µM, selenium 40 mg/mL and sulfur 120 mg/mL after 7 days.
