# Supplementary material for: Selenium, Sulfur, and Methyl Jasmonate Treatments Improve the Accumulation of Lutein and Glucosinolates in Kale Sprouts
Source: Plants (Basel). 2022 May 9;11(9):1271. doi: 10.3390/plants11091271 (PMC9100039; doi:10.3390/plants11091271)

plants

MDPI

Supplementary Materials

Selenium, Sulfur, and Methyl Jasmonate Treatments Improve the Accumulation of Lutein and Glucosinolates in Kale Sprouts

Erika Ortega-Hernández [1], Marilena Antunes-Ricardo [1*], Luis Cisneros-Zevallos [2] and Daniel A. Jacobo-Velázquez [3*]

[1] Tecnologico de Monterrey, The Institute for Obesity Research, Ave. Eugenio Garza Sada 2501, Monterrey, N.L., México, 64849; erika.orhe@gmail.com (E.O.-H.)
[2] Department of Horticultural Sciences, Texas A&M University, College Station, TX 77843, USA. lcisnero@tamu.edu (L.C.Z.)
[3] Tecnologico de Monterrey, The Institute for Obesity Research, Ave. General Ramón Corona 2514, Zapopan, Jal, México, 45201

* Correspondence: marilena.antunes@tec.mx (M.A.-R.); djacobov@tec.mx (D.A.J.-V.).

Figure S1. Seed germination of Red Russian and Dwarf Green kale (*Brassica oleracea* var. acephala) treated with methyl jasmonate, selenium, sulfur or water as control for 7 days

Day	MeJA[1]		
	**25µM**	**50µM**	**100µM**
**3**	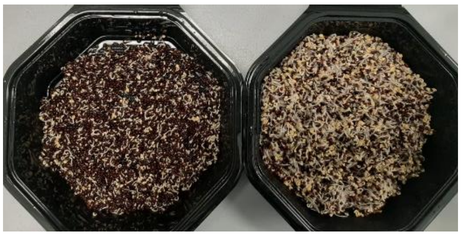	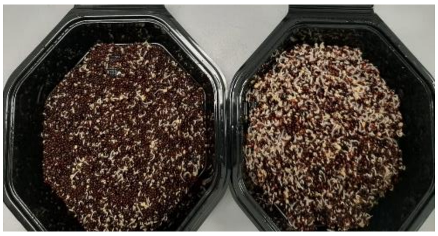	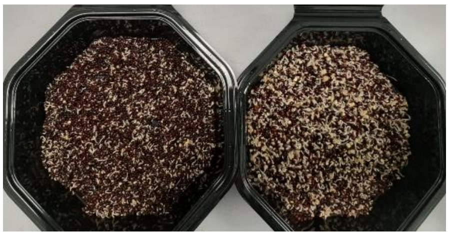
**4**	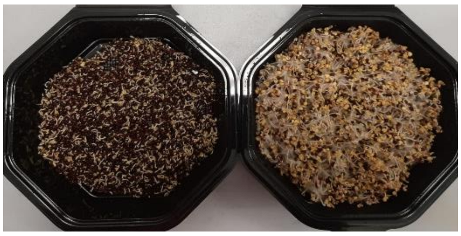	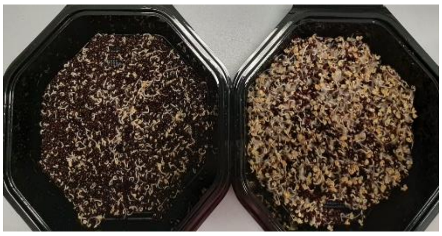	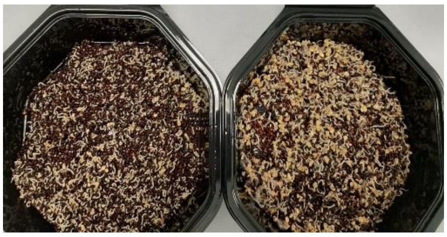
**5**	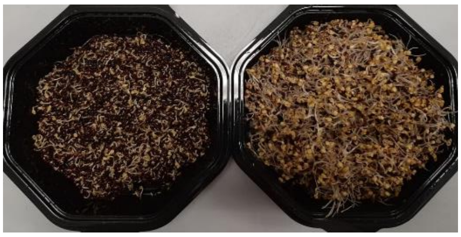	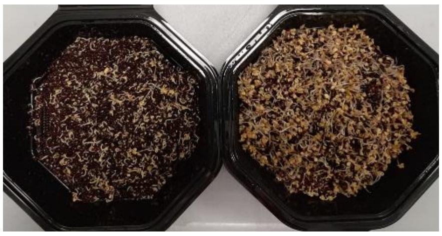	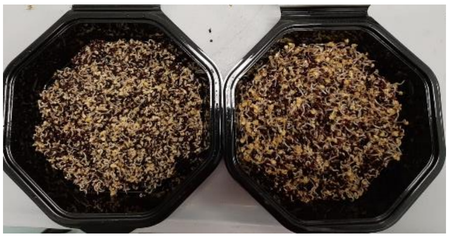
**6**	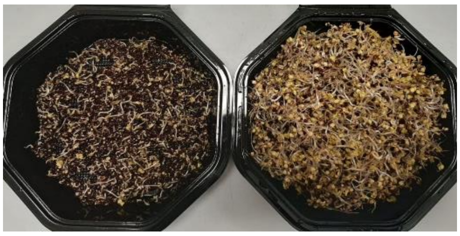	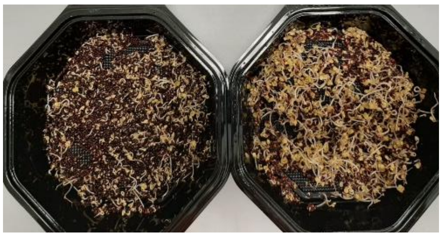	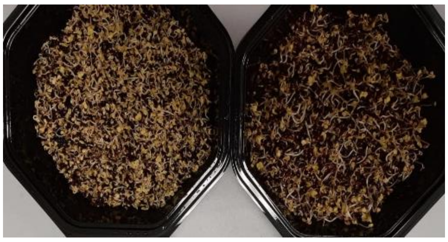
**7**	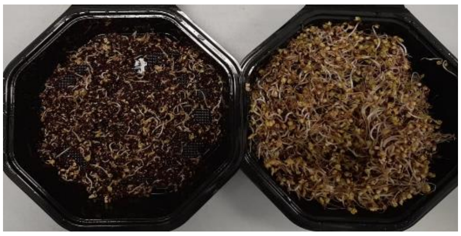	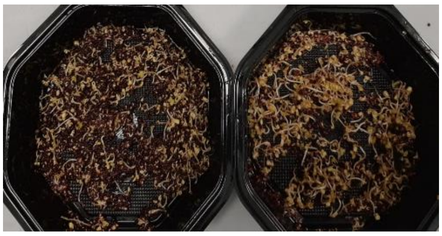	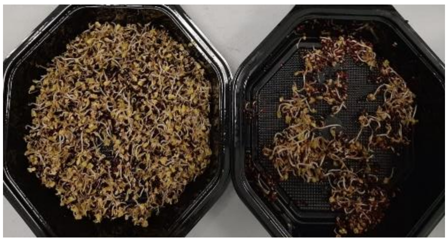

[1] The tray on the left side belongs to the Dwarf Green kale, while the tray on the right side is the Red Russian kale.

Figure S1. (Continuation) Seed germination of Red Russian and Dwarf Green kale (*Brassica oleracea* var. acephala) treated with methyl jasmonate, selenium, sulfur or water as control for 7 days

Day	S[1]		
	30mg/L	60mg/L	120mg/L
3	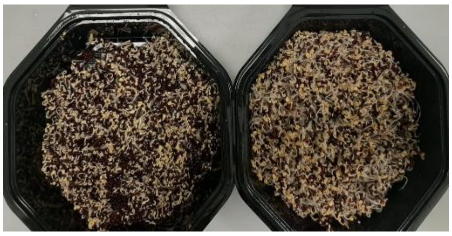	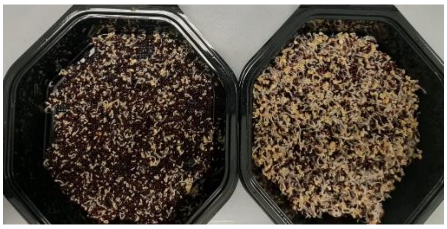	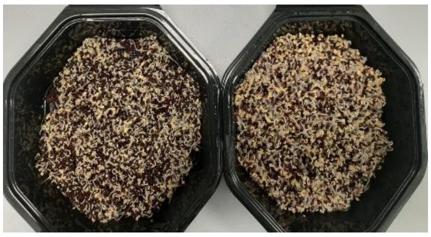
4	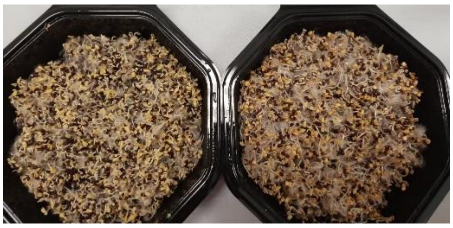	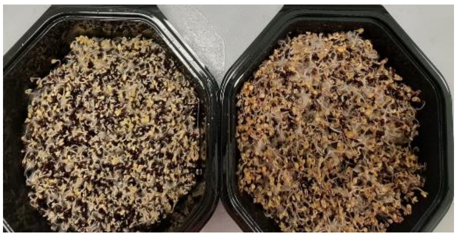	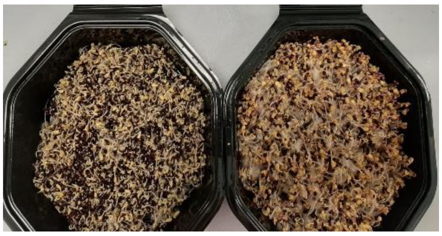
5	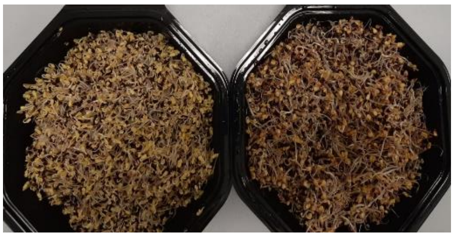	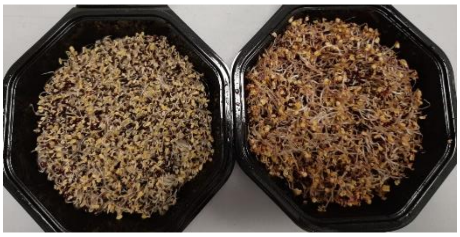	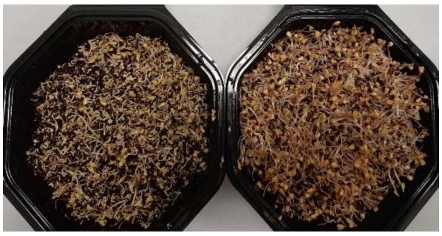
6	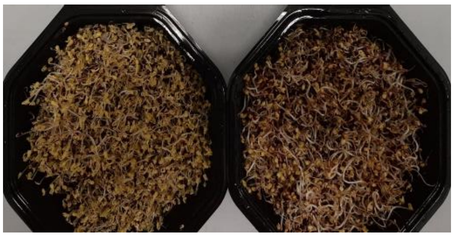	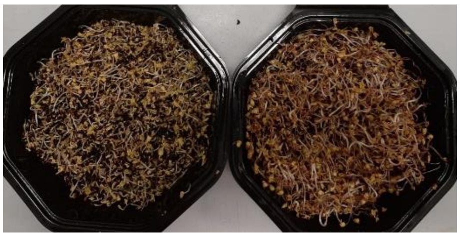	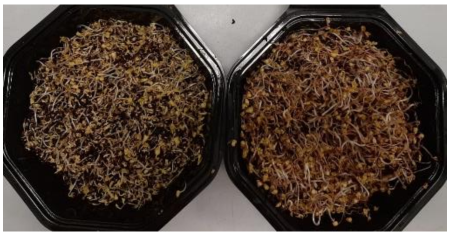
7	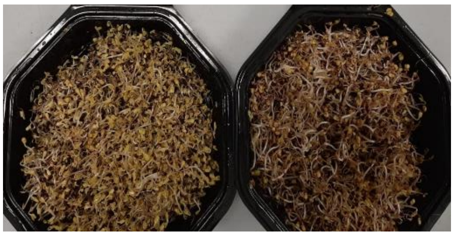	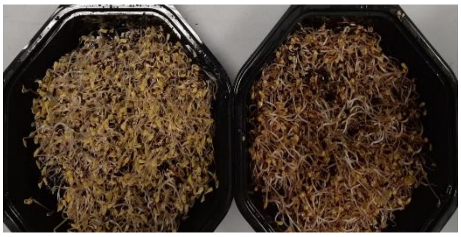	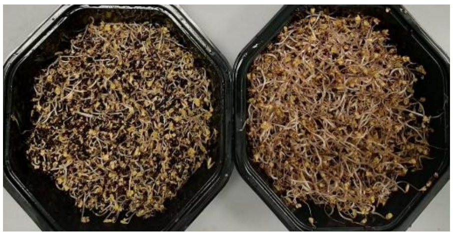

[1] The tray on the left side belongs to the Dwarf Green kale, while the tray on the right side is the Red Russian kale.

Figure S1. (Continuation) Seed germination of Red Russian and Dwarf Green kale (*Brassica oleracea* var. acephala) treated with methyl jasmonate, selenium, sulfur or water as control for 7 days

**Day**		**Se[1]**	
	**10mg/L**	**20mg/L**	**40mg/L**
**3**	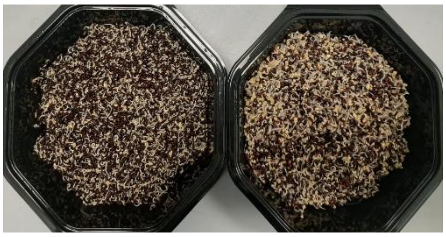	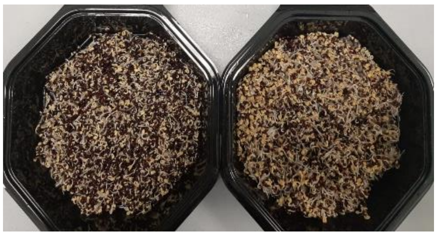	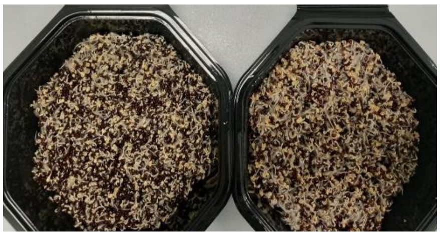
**4**	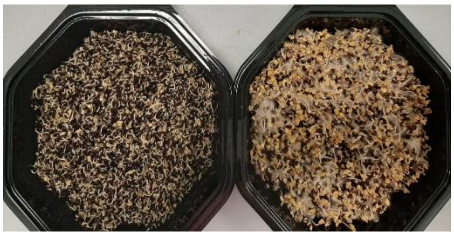	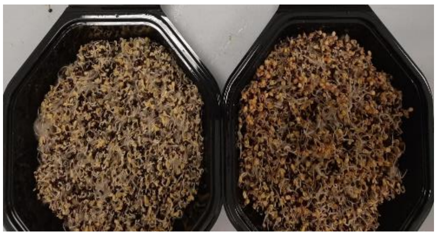	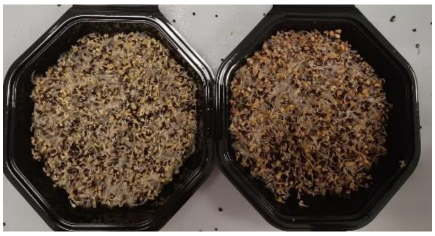
**5**	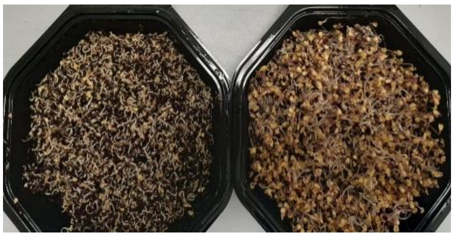	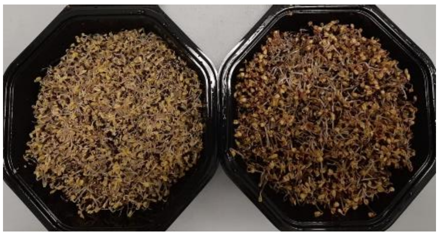	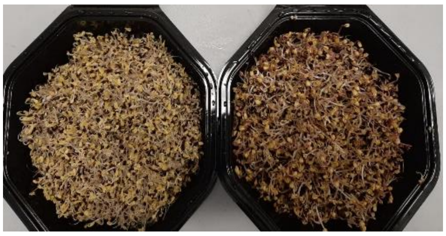
**6**	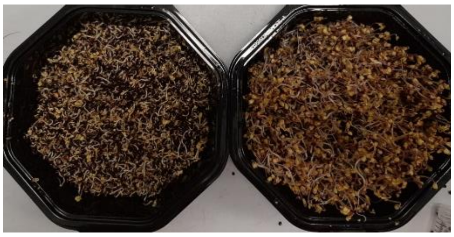	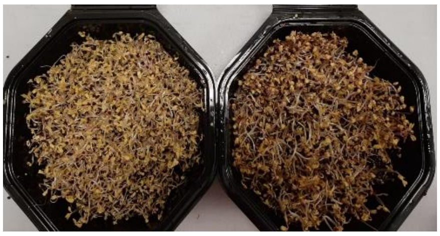	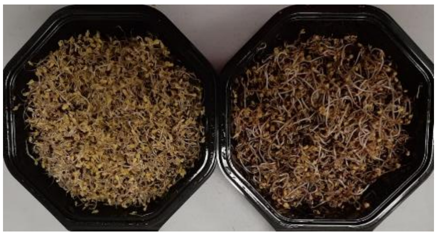
**7**	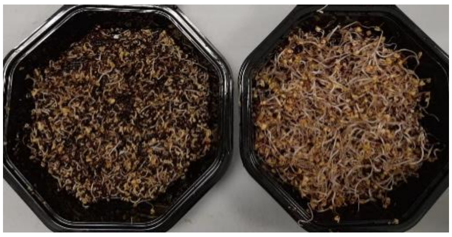	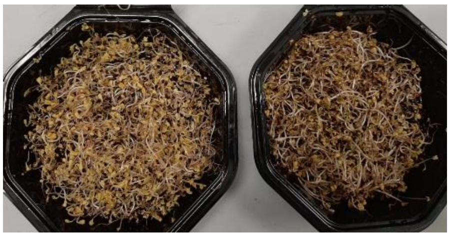	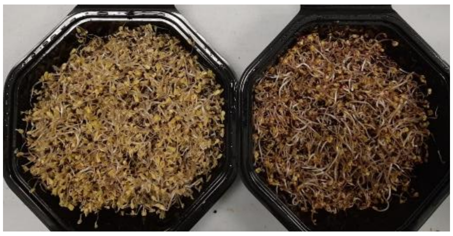

[1] The tray on the left side belongs to the Dwarf Green kale, while the tray on the right side is the Red Russian kale.

Figure S1. (Continuation) Seed germination of Red Russian and Dwarf Green kale (*Brassica oleracea* var. acephala) treated with methyl jasmonate, selenium, sulfur or water as control for 7 days

Day	Control[1]
3	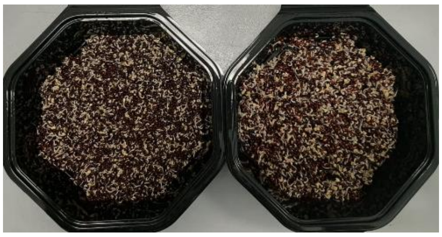
4	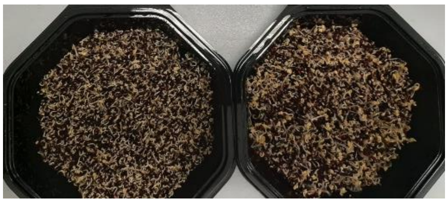
5	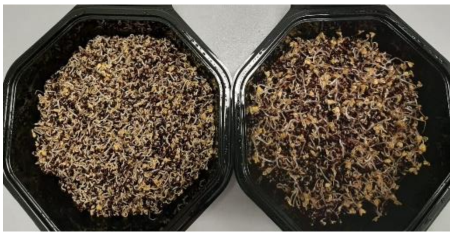
6	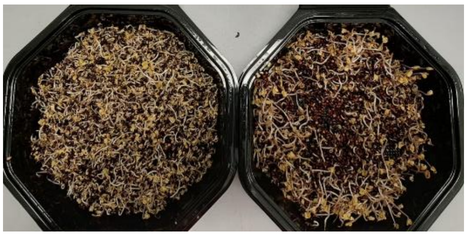
7	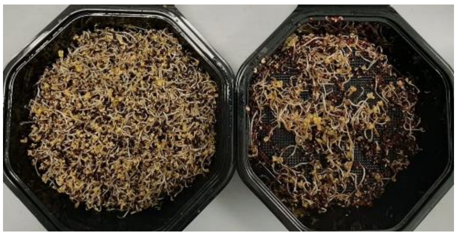

[1] The tray on the left side belongs to the Dwarf Green kale, while the tray on the right side is the Red Russian kale.

Figure S2. Comparison of Red Russian (left) and Dwarf Green kale (right) sprouts (*Brassica oleracea* var. acephala) germinated with most significant treatments: methyl jasmonate 25 μM, selenium 40 mg/mL and sulfur 120 mg/mL after 7 days.

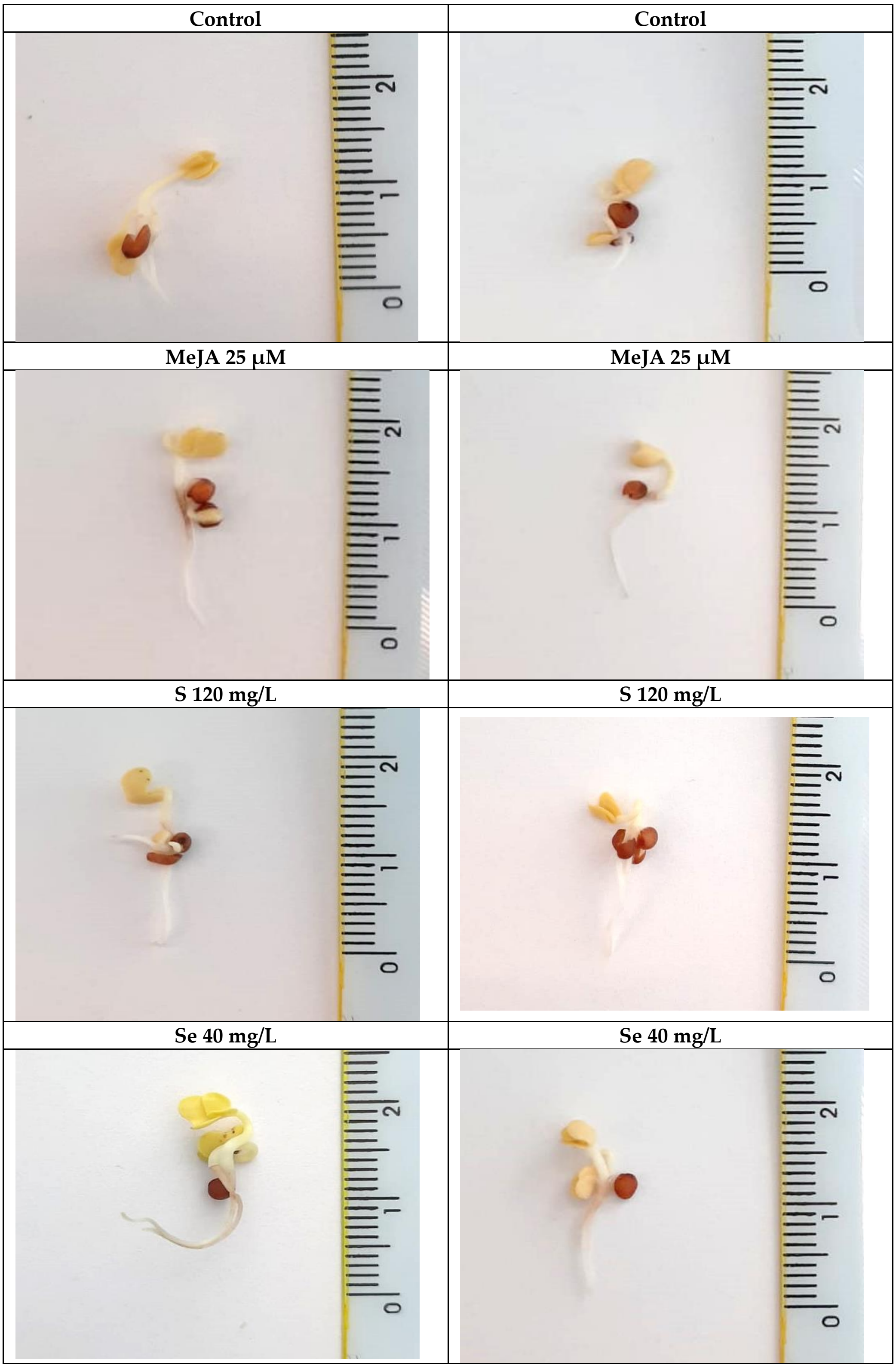

Supplement: Supplementary file 1 [file plants-11-01271-s001.zip › plants-1677205-supplementary.pdf]
